# Senescence Promotes the Recovery of Stemness among Cancer Cells via Reprograming

**DOI:** 10.3390/biom14030288

**Published:** 2024-02-28

**Authors:** Di Wang, Lingbo Liu

**Affiliations:** Institute of Hematology, Union Hospital, Tongji Medical College, Huazhong University of Science and Technology, Wuhan 430022, China; judywang1213@163.com

**Keywords:** senescence, senescence-associated stemness (SAS), cancer stem cells (CSCs), leukemia stem cells (LSCs), stemness reprograming, senescence-associated secretory phenotype (SASP), therapy-induced senescence (TIS), oncogene-induced senescence (OIS)

## Abstract

Both the senescence of cancer cells and the maintenance of cancer stem cells seem to be mutually exclusive because senescence is considered a physiological mechanism that effectively suppresses tumor growth. Recent studies have revealed common signaling pathways between cellular senescence and the maintenance of stemness in cancer cells, thus challenging the conventional understanding of this process. Although the links between these processes have not yet been fully elucidated, emerging evidence indicates that senescent cancer cells can undergo reprograming to recover stemness. Herein, we provide a comprehensive overview of the close correlation between senescence and stemness reprograming in cancer cells, with a particular focus on the mechanisms by which senescent cancer cells recover their stemness in various tumor systems.

## 1. Introduction

Senescence has long been considered a conservative and protective barrier against the uncontrolled proliferation of abnormal cells within the human body. The hallmark of cellular senescence involves cell cycle arrest in response to a sustained DNA damage response after stress damage, therapeutic intervention, oncogene induction, or telomerase dysfunction and can be detected through senescence-related β-galactosidase (SA-β-Gal) [1,2]. This process facilitates DNA repair and prevents the transmission of harmful genetic information to offspring cells. The classic regulatory modes of cellular senescence include cell morphology enlargement, the activation of the tumor suppressor gene *p53*, the upregulation of cell cycle-dependent kinase (CDKs) inhibitors such as p21^WAF1/CIP1^ (CDKN1A) and p16^INK4A^ (CDKN2A), apoptosis resistance, the aggregation of senescence-related heterochromatin (SAHF) within cells, the oxidative stress response, and various metabolic changes. The p53 and Rb pathways synergistically play core roles in these processes. However, in recent years, it was found that the in vitro senescence of cancer cells induced by T-helper-1-cell cytokines can be separate from these two canonical pathways, and displayed a strong dependence on STAT1 and TNFR1 signaling [3,4], thus highlighting the complexity and the multiple regulatory cascades involved in primary driving and other auxiliary regulatory mechanisms.

Another essential feature of cellular senescence is the secretion of the senescence-associated secretory phenotype (SASP), which is mainly composed of various proinflammatory factors, growth factors, and matrix metalloproteinases (MMPs). This process constitutes a highly conserved genotoxic stress response that inhibits further proliferation through cellular autonomous or nonautonomous means, thereby preventing the accumulation of deleterious cells. However, some studies have emphasized the protumor effects of the SASP [5]. We assume that this may be related to the stemness reprograming of SASP in cancers, and this will be elaborated on in the following part.

The senescence inducers of senescent cancer cells mainly involve oncogenes and various therapies, called “oncogene-induced senescence (OIS)” and “therapy-induced senescence (TIS)”, respectively. Although OIS contradicts the description of the abilities to promote proliferation, oncogenes can trigger a robust DNA damage response in cancer cells through excessive proliferation, leading to cellular senescence [6,7,8]. The p53-p21 and p16-Rb pathways have been proven to play important roles in OIS. Meanwhile, therapy-induced senescence (TIS) has also historically been viewed as an antitumor mechanism against the further proliferation and expansion of malignant cells [9]. A large number of treatment methods lead to senescence in cancer cells through genotoxic stress, overactivation of mitotic signals, or oxidative stress. On the other hand, the original definition of cancer stem cells (CSCs) was derived from research on a small group of cells in human acute myeloid leukemia (AML) that can initiate AML in immunodeficient mice after implantation in the body [10], and called them “leukemia-initiating cells (LICs) or “leukemia stem cells (LSCs)”. Subsequently, the existence of cancer stem cells (CSCs) in solid tumor has been confirmed in breast cancer through constructing xenograft mouse models in a similar way [11], these cells can maintain tumors in different cancers during differentiation into pathogenic cells in offspring, thus it is a functional definition. Despite their small proportion among all cancer cells, CSCs contribute significantly to cancer resistance. For instance, leukemia stem cells (LSCs) are believed to be the root cause of refractory/relapse in AML, leading to a poor prognosis. 

Although the majority of stem cells irreversibly undergo differentiation and eventually become specific terminal cells, there is still evidence suggesting that non-stem cell populations can recover stem characteristics, for example, some early progenitor cells in human intestinal tissues exhibit gene expression related to stemness [12,13]. In addition, senescent adult stem cells can directly enhance the reprograming of “Yamanaka factors” (*Oct4*, *Sox2*, *Klf4*, and *c-Myc*) in adjacent cells through the SASP, restoring them to a stem condition with self-renewal and proliferation abilities and maintaining them for two weeks after eliminating the influence of the SASP [14], suggesting that stem cells have high plasticity and can achieve phenotypic reversal. Corresponding to cancers, we make a hypothesis that cancer cells have the ability to reprogram to CSCs and maintain various tumors in vitro and in vivo. In fact, in the past decade, some studies on cellular senescence have supported this hypothesis. For instance, the excessive activation of Wnt-β-catenin signaling in cancer cells with a DNA damage response (DDR) through the p53-p21 pathway can result in stemness characteristics upon release from chemotherapy-induced senescence (described below). In recent years, additional studies have demonstrated the unexpected role of TIS in tumor promotion, particularly in the context of drug resistance in various tumors. In fact, posttherapy drug resistance has become a substantial challenge in cancer treatment strategies [15].

**Figure 1:**
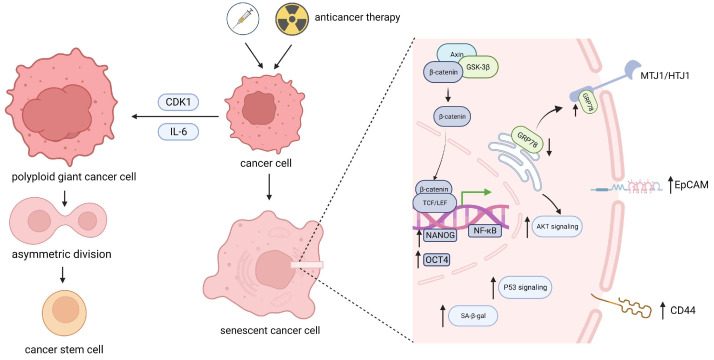
Stemness reprograming in senescent cancer cells. After undergoing anticancer therapy, a portion of tumor cells undergo senescence. These cells can be reprogrammed through increased expression or the corresponding transcriptional regulation of intracellular stem genes such as OCT4 and NANOG or cell surface stem-related markers such as EpCAM and CD44, subsequently affecting downstream signaling pathways. Alternatively, senescence-related CDK1 and IL-6 can induce the production of polyploid giant cancer cells and subsequently proceed via asymmetric division. Ultimately, senescent tumor cells acquire stemness characteristics. OCT4, POU domain, class 5, transcription 1; NANOG, Recombinant NANOG homeobox protein; EpCAM, Epithelial cell adhesion module; CDK1, Cyclin dependent kinase 1; GRP78, Glucose regulated protein 78; MTJ1/HTJ1, DnaJ (Hsp40) homolog subfamily C member 1.

**Figure 2:**
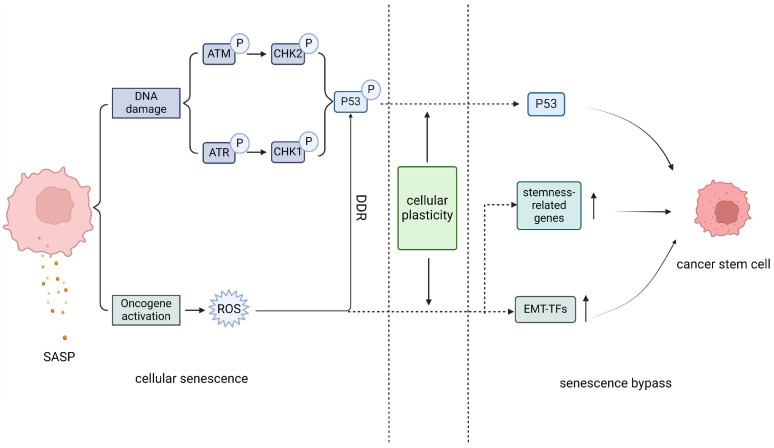
Schematic illustration of senescence bypass. Cellular senescence is characterized by the activation of downstream senescence regulatory molecules caused by a continuous DNA damage response, as well as the excessive activation of oncogenes, which leads to the production of destructive reactive oxygen species in cells. This further maintains the cellular DNA damage response and the cellular senescence state. However, some cancer cell population will choose to experience the senescence bypass due to their highly cellular plasticity, as the part right of the dashed lines, which include changes in the activity of key molecules involved in senescence, upregulation of reactive stem genes, epithelial mesenchymal tendencies, and indirect effects of the heterogeneous SASP, the senescence state gradually weakens. Eventually, cancer cells reverse senescence and transform towards more aggressive cancer stem cells through a pathway distinct from senescence. SASP, senescence-related secretory phenotype; ROS, reactive oxygen species; DDR, DNA damage response; EMT-TFs, epithelial mesenchymal transition-related transcription factors.

Moreover, recent studies have shown that various stem characteristics are enriched in cancer cell populations after TIS or OIS, mainly present as the following aspects: (1) increased expression of stem cell-related markers [16,17,18], such as CD34, CD44, CD133, Lgr6, Nestin, and OCT4 (Figure 1); (2) enhanced expression of proliferation-related markers, such as Ki67, Edu, and ALDH, together with senescence-related markers [19,20]; (3) enhanced tumor formation ability in vitro and increased tumorigenicity in mice in vivo [21]; and (4) CSC remodeling characteristics, including the appearance of epithelial mesenchymal plasticity (EMP) and E/M dual-phenotype subpopulations in CSCs [22]. All of the above findings underscore the link between senescence and the acquisition of CSC properties. In fact, senescence-related stemness (SAS) is emerging as an indispensable factor in cancer resistance. In this review, we aim to elucidate the effects of cellular senescence on stemness recovery in various cancers. Special attention is devoted to the contributing factors and their mechanisms in the acquisition of stemness characteristics among cancer populations. Furthermore, senescence-related stemness reprograming, epigenetic reprograming, changes in metabolic characteristics, and microenvironmental stemness promotion effects represented by the SASP are explored. The goal of this study is to comprehensively understand the phenotypic and mechanistic changes in drug-resistant cancers resulting from senescent tumor cells undergoing genotoxic stress. This understanding will provide a reference basis for targeting these cells in clinical practice and ultimately improve disease prognosis. In addition, recent studies linking senescence to CSC dormancy offer us a new perspective by revealing the unique characteristics of senescent cells, thus suggesting a promising avenue for future research.

## 2. Stemness Reprograming of Cancer Stem cells, as Well as Bypass of Senescence to Promote the Formation of Cancer Stem Cells

### 2.1. Origin and Transformation of Cancer Stem Cells

The irreversible aging and accumulation of somatic stem cells lead to the occurrence of various age-related diseases, including tumors. In the process of tumor occurrence and progression, abnormal tissue-specific stem cells and non-stem cancer cells may contribute to the acquisition of cancer stem cell characteristics [23,24].

#### 2.1.1. Leukemia Stem Cells in Malignant Hematological Diseases

The composition of the blood system perfectly demonstrates the cascade relationship of normal hematopoiesis. Hematopoietic stem cells (HSCs) regenerate themselves and differentiate into multipotent progenitor (MPP) cells, gradually forming hierarchical levels of interrelated daughter cells. Similarly, in AML, LSCs serve as the “root” of cancer cells and continuously differentiate into AML blast cells. However, single-cell sequencing of leukemia patients revealed that 1/2 mutations carried in AML blast cells presented in their normal HSCs [25]. Meanwhile, other research on normal HSCs in AML patients supported that mutations accumulated in normal HSCs can promote their transformation into malignant precancerous cells [10,23]. Although the origin of CSCs remains unclear, Michael F. Clarke et al. reported that CSCs may originate from early progenitor cells. It has also been reported in a previous study with a large number of AML samples that AML-derived LSCs are more closely related to normal progenitor cell subtypes than are HSCs [23]. 

In AML, high-dose cytarabine (Ara-c) induces tumor cell apoptosis [26], while low-dose Ara-c can induce cancer cells to progress to a senescent state. In addition to cytarabine, most chemotherapeutic drugs, including gemcitabine, doxorubicin, cisplatin, and camptothecin [27,28,29], can induce senescence in cancer cells. Most of these agents achieve this effect by inducing DNA damage reactions in cancer cells [30]. In an interesting single-cell sequencing study targeting paired AML patients before and after chemotherapy, it was revealed that compared with that of tumor cells 3 weeks after TIS, a large number of stemness events enriched in recurrent samples after TIS, suggesting that at least some LSCs may be transformed from noncancer stem cells among AML patients who relapse after chemical screening [26]. In addition, this result excluded the possibility of recurrence caused by the amplification of chemotherapy resistant LSCs through detecting drug resistance in chemotherapy residual cells, suggesting that both circulating and quiescent LSCs are highly sensitive to traditional chemotherapy strategies. Contrary to this, previous belief was that LSCs could survive standard chemotherapy methods and cause drug resistance in AML patients after chemotherapy [31,32]. Nevertheless, it is confirmed that pre-leukemia stem cells can survive chemotherapy [33,34,35], although it is currently unclear whether the recurrence of AML can be attributed to further mutations in pre-leukemia stem cells. Furthermore, when AML relapses, LSCs undergo more complex clonal evolution, which may be due to DNA damage caused by chemical treatment. This was also certificated by the high expression of senescence-related genes in the residual AML cell population after chemotherapy [26].

#### 2.1.2. Cancer Stem Cells in Other Solid Tumors

In addition to hematological malignancies, CSCs are present in many other solid tumors, including skin squamous cell carcinoma [36], glioblastoma, breast cancer, and colorectal cancer. There is evidence suggesting that initial carcinogenic mutations occur in stem cells and involve self-renewal, senescence, and apoptotic functions of stem cells, endowing them with the ability to proliferate and expand indefinitely. For example, skin CD34^+^ hair follicle stem cells can evolve into CSCs and progress towards squamous cell carcinoma when p53-mediated cell cycle arrest is lost [37]. Similarly, it was found that the initial mutation of the tumor also occurs in stem cells in human glioblastoma [23]. In addition, in melanoma, JARID1B (a histone demethylase), which mediates the continuous tumor growth, can be induced from negative expression populations [38], indicating that nontumorigenic populations can also exhibit stem cell-like phenotypes.

Furthermore, the phenotype of CSCs is unstable and may be altered or even reversed through cell-cell interactions and complex communication between cells and their microenvironment [39,40]. In fact, CSC subpopulations that can undergo phenotypic conversion have been found in skin squamous cell carcinoma and melanoma [41,42]. Likewise, HIF2a induction, a cellular response to a hypoxic microenvironment, is related to the maintenance of CSCs in glioblastoma and may promote the mutual transformation of non-stem cells (CD133^−^) to CSC-like cells [43]. In breast cancer, CSC-like cells can be regenerated from transformed breast epithelial cells [44]. Moreover, multiple CSC subpopulations may exist in an individual tumor and can clonally reproduce the phenotype of the primary specimen. More intricately, there is heterogeneity among different tumor stem cells, and they also have a high frequency of variations. As a necessary method to verify the function of CSCs, xenotransplantation models demonstrate the multilevel heterogeneity and genetic instability of cancer cells in tumor progression when inducing various cancers [45]. The mechanisms involved in stemness reprograming in CSCs may include signaling pathways such as the Wnt, Notch, c-Myc, Bim-1, and Hdegehog (Hh) pathways, which simultaneously exhibit varying degrees of dependence on different signaling pathways in cyclic and quiescent CSCs.

### 2.2. Stemness Reprograming of Senescent Cancer Cells during Various Types of Senescence

#### 2.2.1. Stemness Reprograming of Senescent Cancer Cells in Malignant Hematological Diseases

The stemness programing of cancer cells after senescence has been confirmed in blood systems. A study of human AML models indicated that, compared with cancer cells that have never been senescent, only senescent cancer cells eventually progress to LSCs with tumor initiation potential, and new AMLs have been established in subsequent mouse transplantation models. Cihangir Duy et al. [26] demonstrated that TIS-induced senescence-like cells with stemness potential are significantly correlated with the NF-κB pathway and can prevent the initiation of AML by inhibiting the upstream signal ATM/ATR of this pathway. Surprisingly, during the process of stem tracking of AML cells in another study, a very small number of aging cells were found to be able to spontaneously acquire stem-related phenotypes without genetic intervention. These findings indicate that during strong genotoxic stress-induced senescence, cancer cells may have some mechanisms to protect themselves from complete clearance but temporarily survive until this toxic environment is exhausted. Although the mechanism of this phenomenon has not yet been studied, it may be due to its secondary role in backing up the reprograming of CSCs [19]. In summary, it is important to mention that CSCs can be transformed from non-stem cells after senescence, suggesting that senescence may serve as a driving factor for the reprograming of CSCs. 

Maja Milanovic et al. examined malignant acute lymphoid leukemia and acute myeloid leukemia models that can induce senescence, and they confirmed that the process by which p53-mediated senescent cells transition from a non-stem state to a stem feature is a result of cellular autonomy rather than SASP mediation [19]. Specifically, non-stem cancer cells that experience senescence rely on the intracellular senescence-related MEK-MAPK and PI3K-AKT signaling pathways to suppress the degradation of β-catenin, a factor downstream of the Wnt protein. Then, glycogen synthase kinase 3β (GSK3β) induces the constitutive activation of Wnt/β-catenin signaling and the expression of its target genes, collectively restoring a stemness phenotype after detachment from senescence shock without affecting Wnt ligand–receptor related activities outside the cell or membrane (Figure 1). This acquisition of a stemness phenotype can be inhibited by specific blockade of the intracellular Wnt signaling pathway, which reveals the mechanism of change in the stemness characteristics of senescent cancer cells. Interestingly, these findings are independent of the conclusion that the SASP can promote tumor cells to obtain stem characteristics through paracrine and/or autocrine effects in the microenvironment, demonstrating the complexity of senescence-associated stemness (SAS) programing for different types of tumor cells in different situations. 

#### 2.2.2. Stemness Reprograming of Senescent Cancer Cells in Other Solid Tumors

More evidence suggests that post-senescence stemness reprograming is also applicable in other solid tumors. It is not unique that after chemotherapy, the expression of stem-related molecules such as EpCAM, CD326, CD24, and NANOG increased in hematological tumors, liver cancer cells [21], pancreatic cancer cells, colon cancer cells, and peritoneal metastasis cells [46] (Figure 1). According to studies of lung cancer model survival after radiation therapy, compared to surviving cells that received the same dose of radiation (6 times), lung cancer cells that survived after 3 doses of radiation therapy strongly expressed stem-related signals, such as CD44, CD133, and OCT4, and were associated with increased senescence-related SA-β-Gal staining [47]. The ALDH^+^ subgroup of senescent pancreatic cancer cells significantly increased after gemcitabine treatment [30]. Significantly, colon cancer cells treated with 5-fluorouracil (5-FU) or irinotecan exhibited an increase in the proportion of lateral group cells, accompanied by a decrease in the proportion of CD44 (mediated colorectal cancer metastasis)-positive cells and an increase in e-cadherin (epithelial marker) levels [48], which may indicate the induction of CSCs and the transition from the mesenchymal phenotype to the epithelial phenotype (MET). Furthermore, in cisplatin-induced lung cancer, stemness reprograming in CSCs is associated with the aging-mediated transfer of glucose-regulated protein 78 (GRP78). It is an important factor located mainly in the endoplasmic reticulum and involves multiple signaling mechanisms, from the endoplasmic reticulum to the cell membrane. During the TIS process, GRP78 accumulates on the cell membrane by anchoring to the transmembrane protein MTJ1/HTJ1, which can activate AKT signaling within CSCs and play a role in regulating stemness [49] (Figure 1). In a lung-specific mouse model, carcinogenic Ras-induced senescent precancerous adenomas can ultimately progress to lung adenocarcinoma, suggesting that a senescent environment may provide favorable conditions for the production of tumor cells and overlap with their key mechanisms. In fact, crosstalk with genes related to the reprograming of CSCs has been found in different regulatory pathways related to senescence [50]. Recently, a large number of papillary renal cell carcinoma samples were examined, and SAS genes highly related to senescence and stemness were screened, including *SIRT3*, *CDKN1A*, *CDK1*, *HSPD1*, *PDGFRA*, *CDKN2B*, *PYCR1*, and *SQSTM1*. These genes were found to predict patient survival [51]. From a mechanistic perspective, the pathways involved in stemness reprograming and the maintenance of CSCs during senescence may include the JAK2/STST3 pathway, the IL-6/STAT3 [52] and NOTCH crosstalk signaling pathways, and the NF-κB/IL-6 signaling axis [53], which are responsible for the generation of CSCs.

### 2.3. Stemness Reprograming of Senescent Cancer Cells via the Division of Polyploid Giant Cells

Polyploidization induction in cancer cells is a characteristic of senescence, especially in OIS. Oncogene-induced senescence (OIS), an anticancer mechanism, is prioritized compared to the transformation of cells to malignant cells induced by oncogenes. However, in the context of OIS, which is thought to cause irreversible cell cycle arrest, RAS activation is still detected in up to 30% of human cancers, which may indicate the existence of other ways that allow some precancerous cells to escape OIS and develop infinite proliferation. In fact, OIS-induced multinuclear giant cells can produce stem cells, similar to highly dedifferentiated tumorigenic cells, through a process similar to asymmetric division, which may represent the early stage of malignant melanoma-derived cells. Asymmetric division of multinucleated cancer cells in polyploid tumor tissue can reduce the fatal risk of DNA damage accumulation in cancer cells [54]. Similarly, this transition from senescence to proliferation through polyploidy is applicable in TIS: chemotherapy induces an increase in the number of stem-like polyploid giant cancer cells, which subsequently undergo asymmetric division, producing cancer cells with cancer-generating potential. Recently, in a study on colorectal cancer, H. Was et al. [48] demonstrated that rare polyploid colorectal cancer cells with blastocyst-like morphology were found in a subset of senescent colorectal cancer cells after chemotherapy, and they may be beneficial for cancer cells in terms of responding to genotoxic stress and continuing to proliferate. In addition, the phenotype of polyploid giant cancer cells in non-small cell lung cancer has been shown to be induced by abnormal expression of CDK1, subsequently preserving the potential of cells to recover DNA replication from senescence through asymmetric division [55] (Figure 1). In ovarian cancer, polyploid giant cancer cells are induced by senescence-related IL-6 and activate the embryonic dedifferentiation program during the initial stage of formation while stimulating normal fibroblasts to reprogram into cancer-related fibroblasts (CAFs) [56].

### 2.4. Senescence Bypass Promotes Stemness Reprograming in Cancer Stem Cells

Recent studies have described how senescent cancer cells bypass senescence-related cell cycle arrest and resume proliferation (Figure 2). Cellular plasticity is considered to occur by reprograming and accumulation at the cellular genomic/epigenetic level after a senescent phenotype is obtained, ultimately leading to the elimination of senescence-related cell cycle arrest. Recent research on acute lymphocytic leukemia (ALL) supports the hypothesis that TIS serves as a necessary condition for driving the stemness recovery of CSCs after the function of p53 is blocked, eventually leading to the initiation of ALL in a mouse model. In the context of pancreatic ductal adenocarcinoma, the carcinogenic *KRAS*-induced benign intraepithelial neoplasia (PanIN) of the pancreas, a precancerous lesion, triggers senescence bypass characterized by epidermal mesenchymal transition (EMT) and upregulation of stem cell-related genes after OIS, thereby allowing cells to transition to stem characteristic cells [57] and completing the necessary steps for the transformation of precancerous lesions to invasive tumors. 

In addition, the induction of senescence may depend on the differentiation status of the cells. Compared to differentiated progenitor cells, CSCs are more likely to evade OIS-induced DNA damage by bypassing senescence through stem cell-related EMT transcription factors (EMT-TFs) [58]. We speculate that although senescence serves as a powerful tumor suppressor, it also confers certain survival advantages on cancer cells that have not yet completed stem transformation, such as through the activation of target involved in stemness-related programs, such as MYC, STAT3, WNT, and NOTCH, allowing them to evade excessive DNA damage during the maintenance of the senescent state, have stronger proliferative abilities after the necessary maintenance conditions are eliminated, and subsequently to refill the human body. This bypass may work as a backup mechanism for the survival and maintenance of CSCs. However, further studies are needed to determine whether the invasiveness of cells caused by this pathway is triggered by complete senescence or driven by mutations in certain oncogenic genes within the cells themselves. In addition, further studies are needed to determine whether these cells that exhibit stem-like features can be considered tumor-initiating cells or cancer stem cells, as currently, these cells are called “stem-like cells”.

In conclusion, the impact of senescence on cancer cell stem reprograming is determined not only by intracellular and intercellular signaling pathways but also by various SAS effects that synergistically promote the invasive development of cancer cells. Cellular senescence occurs throughout the entire human life cycle and is crucial for various physiological processes related to growth regulation [59,60,61], such as embryonic organ development and wound tissue repair. As reflected in findings in tumor systems, Maja Milanovic et al. [62] proposed that the tumor-promoting function of senescence is not a novel regulatory mechanism but rather a “hijacking” mechanism of normal physiological functions to achieve cancer-promoting outcomes. This may reasonably explain the paradox that the inhibitory effect of senescence on tumors and the probability of tumor occurrence increase with age. Many researchers have also proposed that this stemness reprograming may be linked to the ability to survive, driven by the senescent phenotype. This protection mechanism occurs in the event of normal adult cell dysfunction or fatal strikes and is preserved by cancer cells during the transition from normal cells to malignant cells. Hadrien De Blander et al. [58] concluded that SAS may reflect a cellular state in which differentiated terminal cells coexist with poorly differentiated stem cells. Specifically, after experiencing stress, terminal cells that are relatively prone to senescence can escape through polyploid-related dedifferentiation and genomic instability, while undifferentiated cells that are relatively resistant to senescence rely on cellular remodeling to initiate senescence bypass, maintaining the stability of the intracellular genome [63]. These two processes work together to control the response of cancer cells to stress and coevolve. This state of cell reshaping protects the tumor initiation function of CSCs and promotes tumor tissue filling when senility maintenance factors are weakened.

## 3. Epigenetic Reprograming Promotes the Transformation of Senescent Cells into Stem-like Cells 

### 3.1. The Dynamic Remodeling of Epigenetic Modifications in Senescent Cells Contributes to Their Stem Reprograming 

**Figure 3:**
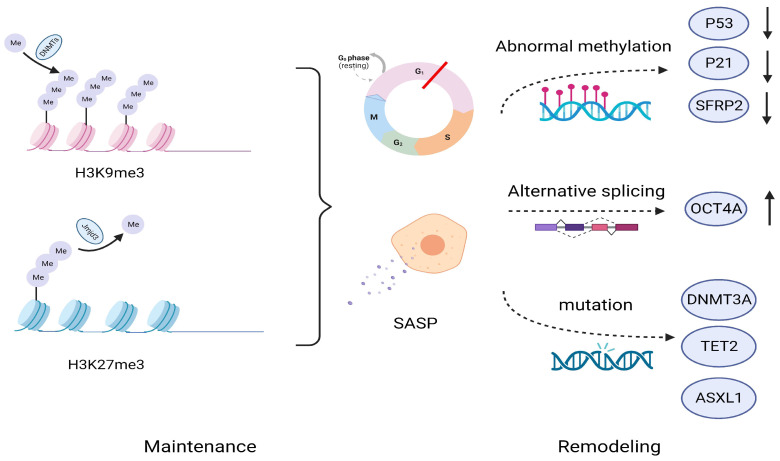
Maintenance and remodeling of cellular senescence at the epigenetic level. Senescence is maintained through epigenetic pathways such as H3K9me3 and H3K27me3, leading to a stable process of cellular senescence, including cell cycle arrest, the secretion of SASP, etc. However, from the perspective of epigenetic variability, these inhibitory chromatin markers may be reshaped over a considerable period of time through abnormal methylation, selective splicing of genes, and mutations in various transcription regulatory factors. H3K9me3, Lys9 trimethylated on histone H3; H3K27me3, Lys 27 trimethylation on histone H3; DMNTs, DNA methyltransferases; Jmjd3, demethylase Jumonji domain containing protein D3; SFRP2, Secret frozen-related protein 2; TET2, ten-eleven translocation-2; ASXL1, ASXL transcribed regulator 1.

The response of cancer cells to the environment regulates the expression of corresponding genes through epigenomic modifications and ultimately determines the fate of cells, such as during cellular senescence [64]. Epigenetic changes are characterized mainly by senescence-related heterochromatin foci (SAHF), which are spatial domains rich in inhibitory chromatin markers. Changes in inhibitory chromatin markers, especially DNA methylation, may be associated with the stemness characteristics of CSCs. The common feature of cell senescence is the stable maintenance of senescence-induced G1 phase cell cycle arrest, especially in OIS and TIS, through the use of the inhibitory trimethylated histone H3 lys9 (H3K9me3), which labels cells near the promoter of cell s-phase-related genes such as Rb (Figure 3). However, this senescent cell cycle arrest controlled by heterogeneous chromatin labelling is replaced by nonmethylated or less methylated H3K9 during the process of cell renewal. In fact, controllable induction or elimination of critical senescence genes such as *Rb* and *p53* can be achieved through epigenetic modifications. Based on these findings, Milanovic M. et al. utilized the important mediating effect of H3K9 methyltransferase (Suv39h1) on Ras-induced OIS and on TIS to design a controllable mouse lymphoma-induced senescence model. By regulating the senescence of mouse lymphoma cells, it was shown that cells undergoing TIS resumed sustained proliferation after removal of doxorubicin (ADR), accompanied by loss of reactivity of SA-β-gal and a reduction in H3K9me3 modification mediated by Wnt signaling. In addition, in recent studies, Patrick M. Perrigue et al. [65] connected the absence of the epigenetic inhibitory marker Lys 27 trimethylation on histone H3 (H3K27me3) in senescence and the activities of its demethylase Jumonji domain containing protein D3 (Jmjd3) and ubiquitously transcribed tetratripeptide repeat, X chromosome (Utx). The deficiency of H3K27me3 during senescence is an obstacle to chromatin remodeling in cancer stem cells. And they demonstrated that after downregulation of Jmjd3 and Utx activities, stem cell-related phenotypes in cancer stem cells can be obtained. In the A549 cancer cell line, senescence-related IL-6 promotes the methylation of p53 and p21 by increasing cellular DNMT1 expression [66], which helps to suppress the weakening of the influence of necessary senescence regulatory factors and promotes the formation of a cancer stem phenotype. Another study suggested that after treatment with etoposide (ETO), PA1 esc-like esophageal carcinoma cells resistant to chemotherapy also exhibited simultaneous upregulation of the stemness-related gene *p21CIP1* and self-renewal-related gene *OCT4A*. This abnormal gene expression pattern involves regular methylation of OCT4A enhancers and subsequent induction of selective splicing in the form of OCT4B, where the proximal and distal enhancers exhibit moderate methylation and unstable expression patterns. However, after cells resumed proliferation, this methylation pattern was largely erased, and the transition of Oct4A partners in the self-renewal and pluripotency network Sox2 and Lin28 was recovered and even enhanced [67]. This finding suggested that some genes in TIS may undergo reversible programing at the expression pattern level, protecting malignant cells from chemical stress.

Notably, epigenetic changes at the cancer cell level are closely related to high remodeling of stem cells, and these two processes may mutually promote. Hazel A et al. [68] performed epigenetic sequencing and observed a certain degree of overlap between senescent cells and cancer cells, which manifested as global hypomethylation and focal CpG island hypermethylation in these senescent cells. These differences are related to the instability of the epigenetic genome in tumors and the transcriptional inhibition of certain tumor suppressor genes, such as *SFRP2*. Interestingly, although the promoter of *CDKN2A* was not detected to contain an increase in the CpG island region, significant increases were still observed in the flanking regions on both sides, indicating that senescence of cancer cells exacerbates the instability of the cell genome and is associated with complex reprograming of cancer cells. This difference may be related to the epigenetic reversibility of senescence and ultimately leads to changes in the internal stem cell characteristics of cancer cells. In summary, due to the strong correlation between cellular senescence and epigenetic changes, as well as the reversible characteristics of epigenetic modifications, theoretically summarizing the irreversible characteristics of senescence is an inaccurate description [69]. Importantly, cellular senescence can facilitate stress conditions that are conducive to epigenetic remodeling.

### 3.2. Epigenetic Remodeling Promotes the Formation and Transformation of Cancer Stem Cells in the Context of Replicating Senescence

Under the premise of replicating aging, the reshaping of various epigenetic landscapes can still promote the formation and transformation of CSCs, which is particularly evident in hematological tumors, especially AML, based on the high incidence and poor prognosis of AML in elderly patients. It has been reported that, compared to those of young AML patients, mutations in epigenetic regulatory factors such as DNMT3A, TET2, SRSF2, and ASXL1 were more common among elderly AML patients and were accompanied by an increase in the frequency of changes in DNA repair- and splicing-related genes [70]. The activity of the important gene *CDC42* in the development of AML increases with the aging of human cells and is associated with impaired function of normal HSCs [71,72]. In addition, it has also been reported that telomere-related dysfunction promotes the occurrence of myelodysplastic syndromes (MDS) in mice by inducing abnormal RNA splicing [73]. In summary, we believe that in the context of aging, the high frequency of epigenetic changes and complex interactions with the genome promote the dysfunction of normal HSCs and the transition to abnormal hematopoiesis, with some driving mutations ultimately leading to the formation of LSCs.

## 4. Changes in Cellular Metabolism after Senescence Promote the Acquisition of Stemness

### 4.1. The Metabolic Characteristics of Cellular Senescence and the Relationship between Stem Cells

Dysfunction of oxidative phosphorylation (OXPHOS) is a very important component of many cellular senescence processes and includes, but is not limited to, decreased activity of the electron transport chain (ETC) within mitochondria, inhibition of mitochondrial respiratory function, and a decrease in membrane potential homeostasis. All these factors are associated with an increase in reactive oxygen species (ROS) within mitochondria. Recent studies have shown that targeting the clearance of mitochondria specifically in senescent cells can reverse the senescence phenotype and rejuvenate cells [74]. In normal tissues, cellular senescence affects the qualities of stem cells and progenitor cells by activating the DDR, subsequently damaging stemness-related genes, and inducing their differentiation program activation. The production of excessive ROS induces stem cell differentiation through oxidative stress; therefore, maintaining low levels of ROS in various body stem cells is crucial for their normal physiological functions. In normal HSCs, fatty acid oxidation (FAO) in mitochondria determines whether stem cells undergo symmetrical division towards differentiation or maintain self-renewal through asymmetric division [75]. CSCs have high levels of mitochondrial metabolism and rely on glycolysis to maintain their low level of ROS, thereby maintaining a stemness state.

### 4.2. Both Mitochondrial Metabolism and Reactive Oxygen Species Regulate Cellular Stemness Characteristics after Senescence

**Figure 4:**
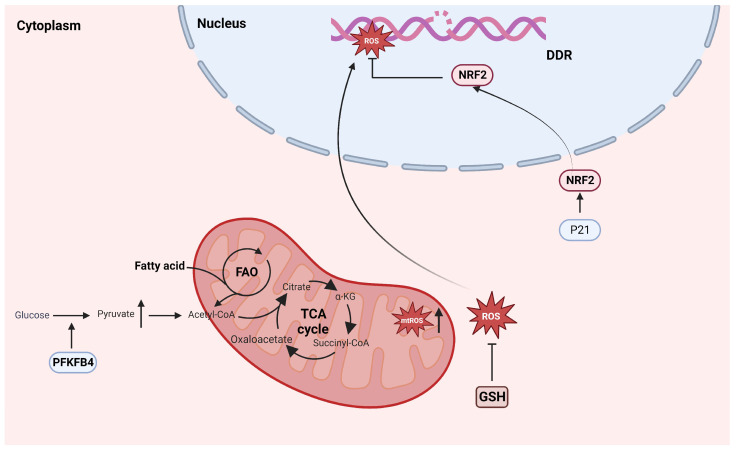
Common characteristics of senescence and cancer stem cell metabolism. Senescence cells produce excessive ROS, promoting genomic instability and exacerbating malignant transformation. In CSCs, p21 neutralizes excessive ROS by increasing NRF2 transcriptional activity and relies on FAO to minimize the impact of OXPHOS. PFKFB4, 6-phosphoFructo-2-kinase/Fructose-2,6-biphasase 4; FAO, Fatty acid oxidation; TCA, tricarboxylic acid cycle; A-KG, a-ketoglutaric acid; ROS, reactive oxygen species; mtROS, mitochondrial reactive oxygen species; DDR: DNA damage response; NRF2: NFE2 Like BZIP Transmission Factor 2; GSH, glutathione; OXPHOS, oxidative phosphorylation.

The low levels of reactive oxygen species (ROS) produced by CSCs and released by senescence, especially mitochondrial ROS (mtROS), may seem contradictory. However, senescence-related mitochondrial dysfunction and increased production of ROS further exacerbate the DNA damage response (DDR) and maintain increased genomic instability [74], leading to dysfunction of normal adult stem cells and possibly providing important conditions for malignant transformation of precancerous lesions and induction of CSCs (Figure 4). The strong instability of the genome is a characteristic of tumors and their stem cells. In fact, according to a recent study about senescence and cancer, genomic instability constitutes a common “meta characteristic” [50]. In AML patient samples, isocitrate dehydrogenase (IDH) is expressed in the early stages of the disease and significantly promotes the development of pre-leukemia [76].

Low level of ROS may be correlated with abnormal intracellular NF-κB signaling in LSCs [77,78,79]. In addition, high mitochondrial metabolic characteristics in LSCs have been reported [75], and glutamine may protect leukemia cells from excessive oxidative phosphorylation by increasing glutathione (GSH) levels and neutralizing excessive ROS. Recently, Haobin Ye et al. [80] demonstrated that LSCs exhibit a subset of proinflammatory phenotypes enriched in CD36 that confer chemotherapy resistance by promoting fatty acid metabolism in cancer cells. CD36 has been shown to be involved in the regulation of the SASP [81]. Furthermore, in a study of ER+ breast cancer, Pingping Shen et al. [82] reported that MCF-7 breast cancer cell lines treated with CDK4/6 inhibitors generally experience senescence and subsequently exhibit increased expression of stemness-related genes. Moreover, they established the relationship between PFKFB4 (a rate-limiting enzyme involved in the cell glycolysis metabolic pathway, with dual functional activities of kinase and phosphatase) and breast cancer cell stemness. The increased expression of PFKFB4 after senescence promotes cell stemness by enhancing glycolysis and pyruvate metabolism in breast cancer cells and reprograming the glucometabolic pathway in cancer cells. Other studies have shown that CD44^+^/CD24^−/low^ stem-like drug-resistant breast cancer cells that survive after neoadjuvant chemotherapy have low levels of ROS, which is mediated by the loss of activity in the TIS-related p21 and 26s proteasomes, leading to increased stability and transcriptional activity of the downstream target protein NRF2 [17] (Figure 4). In recent studies on the effects of chemotherapy on cancer cells, Abisai Dominic et al. observed that anticancer drugs induce the production of mtROS by directly and/or indirectly affecting mitochondrial function, which is also a characteristic of cancer cell senescence. During the senescence of cancer cells, the synthesis of nicotinamide adenine dinucleotide (NAD+) is reduced, and NAD+ is an important mediator of mitochondrial metabolic function. Subsequently, a decrease in sirtuin (SIRT1) deacetylase activity leads to downstream mitochondrial dysfunction and crosstalk with the nucleus, thereby exacerbating DNA damage to the intracellular genome and forming a positive feedback pathway to induce sustained mtROS production [83]. The instability and maintenance of this genome endow CSCs with the ability to acquire value-added driving mutations and generate tumors. Additionally, the overexpression of phospholipase D2 (PLD2) in colorectal cancer cells induces fibroblast senescence in the microenvironment and SASP secretion related to phospholipid acid, activating the Wnt pathway to promote their stemness characteristics (described in more detail below). In this process, senescence acts as a bridge between phospholipid metabolism and cancer stem cell production [84].

## 5. The SASP Reshapes the Stemness Characteristics of Cancer Cells at the Microenvironmental Level

### 5.1. SASP-Mediated Maintenance of CSCs

The microenvironment within tumor tissues has a significant impact on the maintenance and differentiation of CSCs and includes intercellular interactions, secretion factors, the inflammatory microenvironment, the extracellular matrix, and hypoxia signaling [85]. Although the SASP can mediate the recruitment of immune cells to the microenvironment to promote tumor suppression in a short period of time, it seems to be beneficial to the body. In various TISs of cancers after treating, including breast cancer, prostate cancer, lung cancer, ovarian cancer, and malignant mesothelioma, increased levels of proinflammatory factors secreted by the SASP of cancer cells are significantly related to cancer drug resistance and poor prognosis. There is evidence suggesting that CSCs can resist the impact of chemical drugs and promote subsequent drug resistance in tumors, indicating that the senescence-related SASP plays a key role in tumor cell stemness-related remodeling [86]. In addition, the heterogeneity of the SASP produced by senescent cells from different sources increases the complexity of the interaction between the SASP and the surrounding microenvironment, leading to difficulties in accurately defining whether the SASP promotes or inhibits the overall occurrence and development of cancers. In invasive HER2+ breast cancer, IL-6 and IL-8, which are the core inflammatory factors in the SASP, are related to the stemness of CSCs. IL-6 and IL-8 can create a chronic inflammatory microenvironment that allows tumor cells to grow and proliferate through direct and/or indirect effects, including cell–cell interactions or cytokine secretion. These effects change the phenotype and status of tumor cells through cell plasticity. In the breast cancer cell line MCF-7, these effects enhance stemness and promote the tumorigenicity of cells.

From a mechanistic perspective, IL-6 and IL-8 in the SASP work together with tumor cells to induce stromal cells to generate a fibroblast-like morphology, accompanied by the expression and migration of the stemness-related gene *CD44*, as well as the enhancement of self-renewal and multilineage differentiation, suggesting that IL-6 and IL-8 are relevant to the increased stemness of breast cancer cells, regardless of whether they are alone or coexist. Interestingly, this phenomenon simulates the changes in breast cancer cells after senescence and increasing IL-6 and IL-8 levels in the microenvironment results in the “self-circulation pathway” to promote the acquisition of a stemness phenotype in cancer cells. The SASP-related cytokine IL-6 activates the JAK/STAT axis and intracellular NOTCH signaling pathway through paracrine stimulation and intracellular autocrine signaling, thus mediating malignant cell transformation [87] and promoting the acquisition of CSCs characteristics. In addition, IL-8 promotes the self-renewal of breast CSCs by increasing the expression of the CXCR1 receptor [88], and the SASP-related cytokine CCL5 can activate c-Myc and cyclin D1 to promote tumor cell proliferation. Furthermore, in a study of epithelial cell senescence, Brigit Ritschaka et al. [5] reported that primary keratinocytes from mice subjected to OIS induced by the oncogenic gene *HRas^V12^* exhibited increased skin stem cell-related gene expression during brief exposure to SASP, while new keratinocytes did not express stem cell-related markers, indicating that this senescence-related stem marker was induced de novo during the senescence process. Importantly, co-transplantation of newly formed keratinocytes or purified CD34^+^ hair follicle stem cells (HFSCs) that have undergone OIS with dermal fibroblasts into nude mouse wounds can successfully induce the development of large hair follicles at the wound site, demonstrating that keratinocytes that have undergone OIS can perform stem cell-related functions similar to those of HFSCs in vivo. The increased expression of senescence-related stem genes may be attributed to NF-κB-mediated branching rather than through the classical NF-κB pathway, and this change is independent of the cell proliferation phenotype. Importantly, these cell subpopulations did not escape the induction of senescence but expressed stemness-related markers. These cells also expressed high levels of classic senescence markers, such as those of the p53-p21/Rb-p16 signaling pathway, which is different from the findings in some cancer cell populations that can bypass the induction of senescence and volatilization characteristics. This group of cancer cells may not experience “deep” or complete senescence with “potential stemness”; alternatively, there may be specific regulatory mechanisms that differ from cellular signaling pathways that affect cellular senescence and stemness. Interestingly, the analysis of stemness levels after OIS in papillary tumors suggested that the relationship between senescence and stemness is equally applicable in the precancerous state. Importantly, cells with high expression of stem-related markers exhibit increased proliferation and tumor induction ability once P53-mediated cell cycle arrest is lost.

The SASP is also secreted by normal cells, such as stromal cells and fibroblasts, in senescent tumor tissue and has been shown to promote the nonautonomous growth of tumor cells via paracrine secretion. This induction of senescence in the tumor stroma and microenvironment may be physiological or caused by the efflux of senescence factors by cancer cells [89]. In breast cancer cell lines, senescent mesenchymal cells can provide shelter for precancerous cells and cancer cells and promote their progression to cancer cells and malignant status. An analysis of mesenchymal stem cells (MSCs) after cisplatin chemotherapy revealed that MSCs can activate multiple downstream signaling pathways, and the levels of CXCL1, IL-6, IL-8, CCL2, and MIF in the culture medium increased. Additionally, the levels of ALDH and CD24^−^/CD44^+^/EpCAM^+^ cancer stem cell-like cell markers increased in breast cancer cells after chemotherapy [90], which helped cancer cells resist chemotherapy drugs and increase their stemness at the level of cell transcription. This inflammatory microenvironment shaped by senescent cell populations has a role in promoting the progression of various precancerous tumor cells [91]. In the presence of senescent fibroblasts, various precancerous cells can be induced to proliferate and form tumors.

### 5.2. SASP-Mediated Stemness Reprograming

In addition to its impact on the stemness phenotype of tumor cells, SASP-mediated remodeling contributes to stem-like characteristics. In fact, CSCs can simultaneously express both epithelioid- and mesenchymal-related genes and rely on their high plasticity to facilitate invasive phenotype transitions in complex interactions between cells and/or microenvironments [22]. This phenomenon is specifically reflected in epithelial mesenchymal plasticity (EMP) in tumors, which fine-tunes the expression of epithelial/mesenchymal genes in different contexts through the hybridization phenotype of cells to adapt to survival, migration, invasion, and proliferation [92,93]. Studies have shown that IL-8 is associated with the induction of epithelial mesenchymal transition (EMT). In the tumor microenvironment, the IL-8/IL-8R axis is regulated by various signaling pathways, including the NF-κB and Wnt signaling pathways [94], strongly suggesting that the SASP can participate in invasive metastasis and stem cell acquisition in tumor cells. Additionally, Xueqiang Gao confirmed [95] that IL-6 can promote epithelial mesenchymal transformation (EMT) in breast cancer patients. Matrix metalloproteinases (MMPs) in the SASP can synergistically promote epithelial mesenchymal transition (EMT) with HGF, which is beneficial for tumor proliferation. Furthermore, the inflammatory microenvironment shaped by the SASP may further exacerbate the accumulation of random genetic changes within tumor cells and promote genomic instability, which helps tumor cells form heterogeneous cell subpopulations. Due to the differences in the sensitivity of different subpopulations to clinical treatment, drug-resistant subpopulations can be selected, leading to the expansion of abnormal malignant clones.

### 5.3. SASP-Mediated Dual Immune Effects

**Figure 5:**
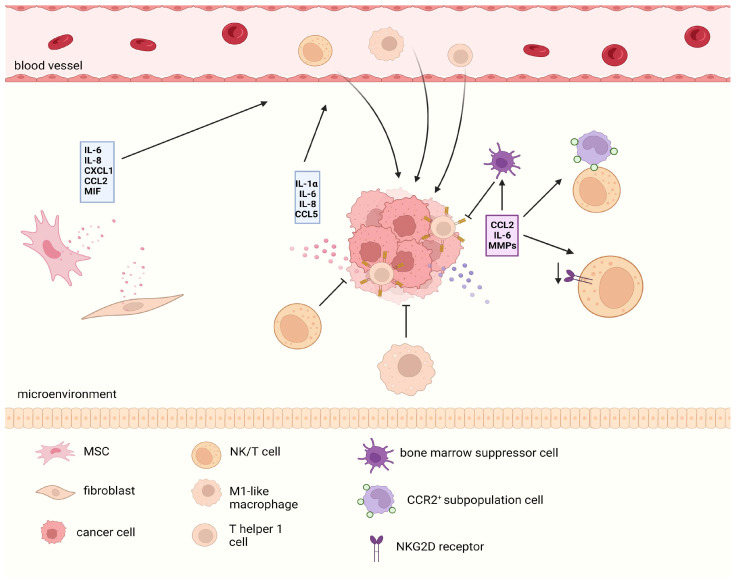
The bidirectional immune effects of the SASP. SASP secreted by various senescent cells in the tumor microenvironment is heterogeneous. On the one hand, they can recruit various immune cells to produce antitumor immune responses. However, in the long term, the SASP can recruit immunosuppressive cells or produce harmful effects by reducing the ligand receptor response related to antitumor immune responses. The red SASP shows a positive effect on antitumor immunity, while the purple part represents a negative effect. SASP, senescence-related secretory phenotype.

It should be noted that the SASP of tumor cells has a bidirectional effect on the recruitment of immune cells into their environment (Figure 5). On the one hand, IL-1α, IL-6, and IL-8 can mediate the recruitment of M1-like macrophages, T helper 1 cells, and natural killer (NK) cells into the tumor microenvironment [96], thus promoting the clearance of senescent cells themselves and exerting a bystander effect in the tumor microenvironment, which may induce non-senescent tumor cells to progress towards a senescent outcome. For example, the use of CDK4/6 inhibitors in melanoma cells can induce the secretion of tumor cells through CCL5, which relies on the NF-κB signal and promotes the recruitment of tumor-infiltrating leukocytes [97], which is the result of stimulating antitumor immunity. However, the response of immune cells to the SASP does not always promote the clearance of tumor cells. The detection of SASP components secreted by MYCN neuroblastoma cells after experiencing different types of TISs revealed that different SASP components have significant differences in terms of the immune effects on tumor cells [98]. Among these agents, low-dose topotecan (TPT) can induce “favorable” SASP and activate antitumor immune responses; in contrast, bromodeoxyuridine-induced SASP secretion by neuroblastoma cells can promote tumor progression in an NFKB1/p50-dependent manner. Moreover, in mouse models of lung- and prostate-derived cancer cell lines, Jana Simova et al. [99] verified that docetaxel-induced senescent cancer cells significantly promoted tumor progression in a mouse model of co-transplantation with non-senescent cancer cells, suggesting that senescent tumor cells exert tumor-promoting effects through the “bystander effect” because the immunosuppressive effect of the tumor cell SASP plays a crucial role. Eliminating this immunosuppression by overexpressing IL-12 (a microenvironmental bridging cytokine that activates NK cells and induces a Th1 immune response) in tumors can restore the sensitivity of prostate cancer to docetaxel. In induced senescent hepatocellular carcinoma, CCL2 attracts a group of CCR2^+^ subpopulations of cells, blocking tumor immune surveillance by binding to NK cells; similarly, IL-6 in the SASP can recruit bone marrow suppressor cells expressing CD11b^+^ and Cr-1 to reduce the immune monitoring effect of antitumor T cells. Recently, in research on the immune clearance of senescent cells in up to 700 patients with prostate cancer or breast cancer, a group of persistent senescent cells was found whom autocrine MMPs could bind to NKG2D ligands in the microenvironment, resulting in the inability to present information to the surface of immune cells, and leading the paracrine pathway to reduce the expression of the NKG2D receptor in immune cells to synergistically escape the immune clearance effect of NK/T cells on tumors in the microenvironment; this phenomenon is particularly significant in OIS [100]. This phenomenon explains why the body still faces the problems of persistent accumulation of aging cells and the formation of a tumor-promoting microenvironment under the immune clearance mechanisms of aging cells. Senescent tumor cells can still escape immune clearance and persist through certain mechanisms. Furthermore, the SASP may be related to the dormancy state of CSCs and will be discussed below.

In summary, these mechanisms summarize the complex regulation of the tumor phenotype by the SASP and may be associated with the type and progression of cancer, different responses of cancer to antitumor immunity, senescence induction methods, and duration. This may involve the stimulation intensity of certain SASP components on tumor cells, as well as the varying strength of connections between various senescence and stem signaling axes. Of note, evidence demonstrated the composition and content of SASP varies at different senescence stages through proteome analysis, and always contained core components of both antitumor and protumor [101]. It may produce antitumor or protumor effects for the development of cancer through stage- and context-dependency, indicating that dynamic monitoring of SASP at different senescence stages can help reveal its complex effects on cancer progression. Given the complexity of the SASP produced by tumor cells, combination therapy to induce tumor senescence and subsequently clear the SASP has been proposed for clinical use and is expected to become a treatment method for clearing tumor cells, especially cells that are resistant to chemotherapy [102,103,104].

## 6. The Senescence of Cancer Stem Cells May Serve as a Dormancy Mechanism to Protect Them from the Negative Impact of Genotoxicity

The dormant state of CSCs is regarded as a quiescent, reversible, and nondividing stem cell state that is largely similar to cellular senescence, especially after the view that cellular senescence has always been considered a stable and irreversible state was challenged. Long-term dormant CSCs can still be activated and cause local or distant tumor recurrence after many years of anticancer treatment, such as the recurrence of breast cancer. Quiescence is considered the main cause of drug resistance in CSCs, at least for LSCs. LSCs can induce dormancy characterized by quiescence when subjected to therapeutic shock, which is reflected in the upregulation of the expression of the antiapoptotic protein Bcl-2 and changes in intracellular metabolic dependence from glycolysis to mitochondrial oxidative phosphorylation (OXPHOS) [105]. Cancer niches have significant implications for the dormancy of CSCs, possibly induced by adjacent cells or their own secretion. AML cells prioritize customization to the endothelial zone of the bone marrow and subsequently reshape the bone marrow microenvironment, leading to abnormalities in vascular structure and changes in the phenotype of bone marrow mesenchymal cells. These changes further lead to the sacrifice of normal hematopoietic function in HSCs and the static maintenance of LSCs [106]. Although research on how the bone marrow microenvironment affects the dormancy function of LSCs is insufficient, it can be confirmed that remodeling of this microenvironment has a fatal impact on normal HSC function.

Although senescence and dormancy are generally believed to occur independently of each other in CSCs, the underlying mechanisms overlap [107] and are correlated. Studies have shown that the CDK4/6-p28 α/β pathway and Ras-MEK-ERK1/2 strongly correlate with dormancy, and these pathways are also crucial for cellular senescence. The presence of TGF-β family members in the SASP has been proven to be important for dormancy, and they were found in the tumor stem cell niche with abnormally increasing concentrations, suggesting their importance in CSCs. In addition, MMPs and VEGF in the SASP can affect the microenvironment of tumor cells, regulate blood supply, and promote the recovery of dormant tumor cell function [108]. In their single-cell RNA-sequence analysis of AML patients, Changir Duy et al. [26] reported that the transcriptional regulation of leukemia cells after TIS is highly similar to embryonic diapause and characterized by senescence-induced depletion of MYC. They also found a high enrichment of LSC-related genes in relapse samples after chemotherapy, thus indicating the correlation between the two. Recently, research identified a detection method of SA-β-gal combined with Ki67 (a marker of cycling cells) and phosphorylation RPS6 (pRPS6, an indicator of active protein synthesis) for distinguishing between cellular senescence and quiescence. The principle is that the proliferation and protein synthesis of quiescence cells exhibit dual low characteristics, while senescent cells still maintain their state of synthesizing various proteins despite lacking proliferation ability, thereby sustaining the senescence phenotype [109]. 

Regarding the correlation between senescence and dormancy in CSCs, Tareq Saleh et al. [108] believe that senescence is more suitable for describing tumors entering a dormant state than for describing tumors entering a quiescent state, as senescence involves more genetic modifications and morphological changes that are not present in a transient quiescent state. However, Francisco Triana Martinez et al. speculated that there are two types of dormancy characteristics within cancer cells: short-term dormancy characterized by quiescence and long-term dormancy characterized by senescence, and that the choice of these two modes depends on the origin of the cell and its ecological niche [110].

## 7. Discussion

Senescence is not the ultimate state of the cell population but rather an intricate and fragile process regulated by diverse pathways and various mechanisms requiring coordinated maintenance both intracellularly and extracellularly. The variability of the cell cycle arrest state is likely linked to the degree of cellular senescence and is regulated by certain core signaling pathways. The role of CSCs in tumor initiation and drug resistance cannot be ignored in the context of different senescence types, emphasizing the need for further research on the interplay between cellular senescence and stem reprograming, and the proposal of the SAS mechanism also supports this view. Understanding the connections between cellular senescence and the stemness of CSCs involves a nuanced exploration of the relationship between replicative senescence, stress-induced senescence (TIS), and oncogene-induced senescence (OIS). Different types of senescence may represent variations in the depth of senescence induction, among which cells with insufficient relative senescence depth are endowed with greater survival abilities. Therefore, investigating the sensitivities of SAS to different types of cellular senescence can elucidate the mechanisms underlying this phenomenon.

The degree and pattern of chromatin remodeling emerge as crucial factors influencing the stability of the senescent state, particularly cell cycle arrest. Although evidence linking chromatin remodeling to metabolic reprograming and dormancy in cancer cells is scarce, a paradoxical relationship between the metabolic characteristics of senescence and stemness complicates the picture. The previously assumed lack of correlation between senescence and dormancy is also worth revisiting in this context.

The SASP plays a pivotal role in CSCs acquisition through various processes, forming a significant component of the SAS. The stemness enhancement effect mediated by the SASP involves the generation of a proinflammatory microenvironment, intercellular secretion and signal transduction, an impact on tumor stromal cells, and immunosuppressive effects. In addition, the role of the SASP in reprograming the metabolic and dormant characteristics of CSCs has also been demonstrated, and in a collaborative manner, it promotes the acquisition of CSCs.

Due to the discovery of the SAS in various tumors, researchers have proposed a “pathway indifference” theory [111]. It is assumed that the population resistance of certain cancers is not solely caused by the activation of carcinogenic pathways but also relies on specific phenotypes that promote cell survival to confer resistance, such as post-senescence SAS and adaptive cloning. This phenomenon may also explain why blocking fragile targets of drug resistance signals alone cannot completely eliminate this disease.

One limitation is whether the changes in the stem cell characteristics of cancer cells after senescence are caused by cancer cell reprograming or by genotoxicity screening after DNA damage; subsequently, the amplification of stem cells with anti-senescence cycle arrest was selected. This may require targeted tracking and lineage analysis of senescent cancer cells at the single-cell level [112] to obtain a more convincing description and reveal the heterogeneous subpopulation characteristics of CSCs. This should also be conducted on AML patient samples. However, there is also evidence that the stemness feature reprograming of CSCs in triple-negative breast cancer is the result of “competitive release” caused by chemotherapy [113] due to the strong environmental adaptability of this rare drug-resistant subgroup. In addition, due to the heterogeneity of senescence and the lack of widespread adoption of single-cell omics, current detection of cellular senescence still mainly relies on SA-β-gal [114], which is greatly influenced by the PH value of the external environment, thus false positives are possible. This increases the difficulties of accurate detection of cellular senescence. Hopefully, it may be feasible to cooperatively detect senescence combined with stemness phenotype. Another limitation is that in complex organisms, long-term senescence can promote the development of cancer cells towards greater value-added invasion. However, it is not possible to describe the impact of cancer’s cellular senescence in a single way to define whether senescence is beneficial or harmful at a certain time afterwards, which increases the difficulty of developing clinical medication targeting cellular senescence. Furthermore, the description of “cancer cell stemness recapture” is inaccurate. In fact, “stemness” involves many phenotypes, such as self-renewal and continuous division. In addition, heterogeneity within CSCS limits the comprehensiveness of the current description of tumor cell stemness characteristics. We prefer to define these cells as “stemness-like cells”. A generalized description of stem characteristics may be able to fill this gap in the literature through single-cell multiomics techniques such as single-cell transcriptomics combined with spatial transcriptomics [115]. Finally, SASs occur mainly in TISs and OISs and are caused by intense stress on cells, leading to a strong DDR. This process is not identical to replication aging characterized by telomere dysfunction. Therefore, exploring the long-term effects of senescence on cancer development in complex organisms necessitates expanding the exploration of SAS from tumors to a broader range of ageing diseases. Recently, studies have shown that the SAS plays an important role in patients with atherosclerosis [20].

Given that cancer cells still have the potential to restart tumors after acquiring a senescent phenotype, it is necessary to target the remaining senescent cancer cells after therapy. A sequential combination therapy method called “one-two-punch” has been proposed to target the acquired defects or vulnerability of senescent cancer cells, including senolytics targeted to senescent cells and senomorphic to the SASP [1,69]. However, the limitations of this therapy must also be recognized. First, there are currently no extensive clinical cases of using sequential therapy to combat cancer cells. Second, it is not yet known which treatment regimen will achieve better survival outcomes than previous treatments that induce the majority of cancer cells to undergo apoptosis through chemotherapy. Another treatment strategy is to target cells strongly affected by SAS through the widespread use of precision medicine for researching tumors at the single-cell level, which can reveal the vulnerability of SAS subpopulations. Finally, a strategy proposed in clinical practice aims to pursue a stable senescent state of cancer cells rather than completely eliminating them to further induce tumor cells with a restored cell cycle to a more complete and non-proliferative state or to selectively passivate the SASP [116], thereby reducing the effect of cancer stem cell reprograming.
